# Hazelnut Pollen Phenotyping Using Label-Free Impedance Flow Cytometry

**DOI:** 10.3389/fpls.2020.615922

**Published:** 2020-12-08

**Authors:** Lorenzo Ascari, Valerio Cristofori, Federico Macrì, Roberto Botta, Cristian Silvestri, Tommaso De Gregorio, Eloy Suarez Huerta, Marco Di Berardino, Silvan Kaufmann, Consolata Siniscalco

**Affiliations:** ^1^Department of Life Sciences and Systems Biology, University of Turin, Turin, Italy; ^2^Department of Agriculture and Forest Sciences, University of Tuscia, Viterbo, Italy; ^3^Department of Agricultural, Forest and Food Sciences, University of Turin, Turin, Italy; ^4^Ferrero Hazelnut Company, Ferrero Trading Lux, Findel, Luxembourg; ^5^Amphasys AG, Technopark Lucerne, Root, Switzerland

**Keywords:** *Corylus avellana* L., cultivar selection, sterile pollen, pollen hydration, pollen viability, reciprocal chromosome translocations

## Abstract

Impedance flow cytometry (IFC) is a versatile lab-on-chip technology which enables fast and label-free analysis of pollen grains in various plant species, promising new research possibilities in agriculture and plant breeding. Hazelnut is a monoecious, anemophilous species, exhibiting sporophytic self-incompatibility. Its pollen is dispersed by wind in midwinter when temperatures are still low and relative humidity is usually high. Previous research found that hazelnut can be characterized by high degrees of pollen sterility following a reciprocal chromosome translocation occurring in some cultivated genotypes. In this study, IFC was used for the first time to characterize hazelnut pollen biology. IFC was validated via dye exclusion in microscopy and employed to (i) follow pollen hydration over time to define the best pre-hydration treatment for pollen viability evaluation; (ii) test hazelnut pollen viability and sterility on 33 cultivars grown in a collection field located in central Italy, and two wild hazelnuts. The accessions were also characterized by their amount and distribution of catkins in the tree canopy. Pollen sterility rate greatly varied among hazelnut accessions, with one main group of highly sterile cultivars and a second group, comprising wild genotypes and the remaining cultivars, producing good quality pollen. The results support the hypothesis of recurring reciprocal translocation events in *Corylus avellana* cultivars, leading to the observed gametic semi-sterility. The measured hazelnut pollen viability was also strongly influenced by pollen hydration (*R${}_{\text{adj}}^{2}$* = 0.83, *P* ≤ 0.0001) and reached its maximum at around 6 h of pre-hydration in humid chambers. Viable and dead pollen were best discriminated at around the same time of pollen pre-hydration, suggesting that high humidity levels are required for hazelnut pollen to maintain its functionality. Altogether, our results detail the value of impedance flow cytometry for high throughput phenotyping of hazelnut pollen. Further research is required to clarify the causes of pollen sterility in hazelnut, to confirm the role of reciprocal chromosome translocations and to investigate its effects on plant productivity.

## Introduction

Hazelnut (*Corylus avellana* L.) is a diploid 2n = 2x = 22 (Guo et al., 2009; Falistocco and Marconi, 2013), self-incompatible, monoecious species with peculiar reproductive biology, being wind-pollinated and flowering during late winter or early spring (Germain, 1994). Its cross-pollination is enhanced by the sporophytic self-incompatibility occurring at the stigmatic surface, which is controlled by a single locus with multiple alleles (Mehlenbacher, 2014). Fertilization is strongly delayed and pollen tubes wait dormant for weeks at the base of the style for ovules to be fully developed (Liu et al., 2014). The increased economic interest toward hazelnut has brought its cultivation in new areas, where plants are often subjected to different environmental conditions if compared with those found in the native range. Besides, the lack of wild hazelnuts in these areas can hinder fertilization and cropping without the adoption of proper pollination strategies (Ascari et al., 2018).

To deepen the knowledge about pollination dynamics and improve crop yield, basic and applied research was recently carried out. In particular, the effect of temperature on hazelnut pollen storage (Ellena et al., 2014; Novara et al., 2017), the influence of spermidine and temperature on pollen performance (Çetinbaş-Genç et al., 2019, 2020), and the efficacy of artificial pollination in constrained growth conditions (Ascari et al., 2018) were investigated.

*Corylus avellana* pollen is binucleate at dispersal (Me et al., 1983; Heslop-Harrison et al., 1986; Tiyayon, 2008) and has generally been considered long-lived (Heslop-Harrison et al., 1986). In fact, pollen remains viable for several weeks at +4°C, but quickly dies at +20°C in some cultivars, emphasizing the importance of lower temperatures for keeping pollen viable over the time necessary to land on a compatible stigma (Novara et al., 2017). Moreover, some authors described hazelnut pollen as partially hydrated, having a water content at dispersal higher than 30% (Nepi et al., 2001). In partially hydrated or desiccation sensitive pollen, water loss cannot be regulated, implying that transport to a receptive stigma must be quick enough to keep pollen alive (Franchi et al., 2002; Pacini et al., 2006). Hazelnuts have female inflorescences with dry stigmas (Ciampolini and Cresti, 1998) where a specific pollen-stigma interaction must occur to mobilize water and nutrients toward the pollen grain (Edlund et al., 2004). Taken together, those findings support the hypothesis that environmental temperatures and pollen hydration status play an important role in the reproductive success of hazelnut.

Furthermore, some hazelnut cultivars were found heterozygous for a reciprocal chromosomal translocation, resulting in the production of sterile pollen (Salesses and Bonnet, 1988; Frenguelli et al., 1997; Marinoni et al., 2018). Plants with heterozygous reciprocal translocations usually undergo either alternate or adjacent meiotic segregation, producing 50% of unbalanced non-viable gametes and 50% of balanced normal gametes (Ray et al., 1997). *C. avellana* × *Corylus americana* hybrids also presented irregularities at meiosis in pollen mother cells and partial or total male sterility (Mckay, 1965). Besides, abundant sterile pollen was detected in the cultivar “Tonda di Giffoni” but not in wild hazelnut plants (Ascari et al., 2020). Therefore, hazelnut can be considered a valuable example for investigating the effects of environmental and genetic factors on pollen phenotypes.

Pollen grains with anomalous morphology are usually produced by genomic backgrounds or environmental stressors that hinder pollen development. Because it lacks essential cellular structures, this pollen is unable to accomplish fertilization even in favorable conditions and is considered sterile. Pollen that undergoes a successful development shows a regular morphology and is potentially fertile. Nevertheless, adverse conditions that decrease its viability can occur throughout or after anthesis (Ascari et al., 2020).

Various techniques have been employed for the quantitative analysis of pollen viability and sterility. Some of them require the labeling of pollen grains and the non-automated or automated analysis of micrographs (Mudd and Arathi, 2012; Tello et al., 2018; Ascari et al., 2020). Flow cytometry using fluorescent probes has also been used for the evaluation of pollen DNA content and its response to temperature stress (Kron and Husband, 2015; Luria et al., 2019). Moreover, other authors proposed methods for pollen analysis based on its dimensions (Kelly et al., 2002; De Storme et al., 2013).

Recently, label-free impedance flow cytometry (IFC) was demonstrated as a useful alternative to the above-mentioned techniques for the analysis of microspore development and characterization of pollen viability in different plant species (Heidmann et al., 2016; Heidmann and Di Berardino, 2017; Canonge et al., 2020; Impe et al., 2020).

**FIGURE 1:**
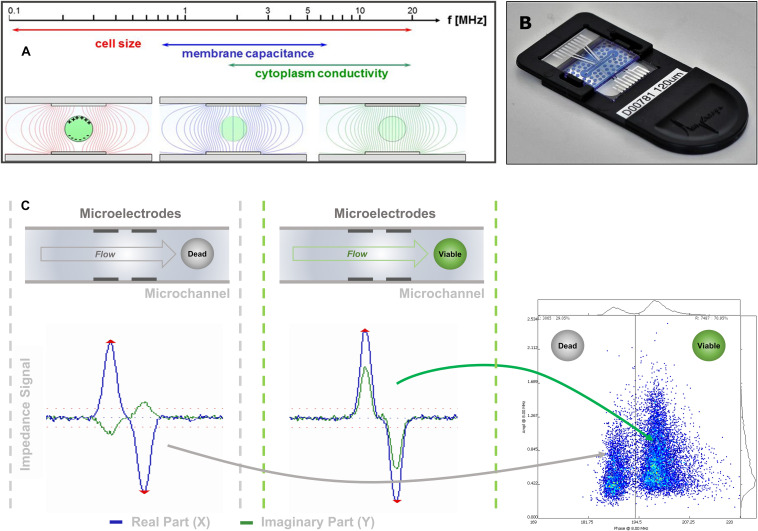
IFC signal acquisition and transduction. **(A)** Due to their dielectric properties, biological cells show a distinct behavior in an electric AC field, which depends on the applied frequency. **(B)** Image of one of the microfluidic chips used in this study. The sensing channel had a dimension of 120 μm × 120 μm. **(C)** The impedance signal obtained from a cell flowing through the microfluidic channel and crossing the electric AC field applied on the microelectrode pairs is split into its real (resistance) and imaginary (capacitive reactance) components. As the capacitive component is dependent on the membrane integrity of the cell, viable and dead cells can be discriminated mainly in their imaginary component if measured at the appropriate frequency.

In IFC (Figure 1), pollen grains in suspension flow through a microchannel, where an alternating electric field is applied. Each pollen grain changes the measured impedance signal depending on its dielectric properties (Sun and Morgan, 2010). At low frequencies (<1 kHz), the phospholipid bilayer of the outer membrane becomes polarized, thereby obstructing current flow and acting as a capacitor (Azzarello et al., 2012). At this stage, information about pollen number and volume can be extracted. At intermediate frequencies (1–8 MHz), membrane polarization decreases and its capacitance and conductance can be characterized, giving information about viability (Figure 1A). Finally, at higher frequencies (>8 MHz), the plasma membrane is no longer an impediment to the electric field and the cytoplasm and organelle status can be interrogated (Cheung et al., 2005).

In this study, impedance flow cytometry was employed to address some of the open questions about hazelnut reproductive biology and pollination and to build a robust protocol for the high-throughput phenotyping of hazelnut pollen. To accomplish this goal, (i) the ability of IFC to measure pollen viability was validated, (ii) impedance was monitored during a prolonged pollen hydration period to define the best hydration period for hazelnut pollen, and (iii) pollen viability and sterility were assessed in an ample set of cultivated and wild hazelnut plants.

## Materials and Methods

### Impedance Flow Cytometry

Impedance signals of pollen in suspension were measured using a commercial impedance flow cytometer Ampha^®^ Z32 (Amphasys, Switzerland). The instrument evolved from the work of Cheung et al. (2005) and can measure up to four different frequencies simultaneously in a 0.3–30 MHz range, using microfluidic chips with parallel facing electrodes and channel sizes of 15 up to 400 μm (Figure 1B). In its simplest form, the equivalent electric circuit of living pollen in an electric field is made of a resistor, the cytoplasm, in series with a capacitor, the plasma membrane (Foster and Schwan, 1989). Impedance extends the notion of resistance to alternate current (AC) and is defined as the voltage to the current ratio (Sun and Morgan, 2010). In cartesian coordinates, impedance Z(*ω*) can be described as the vector sum of a real component Z_r_ (*ω*), or resistance, and an imaginary component Z_i_ (*ω*), or capacitive reactance (Azzarello et al., 2012):

(1) $$Z_{r}\,\left(\omega \right)=\left|Z\right|\,c\,o\,s\,\left(\theta \right)$$

(2) $$Z_{i}\,\left(\omega \right)=\left|Z\right|\,s\,i\,n\,\left(\theta \right)$$

Phase angle (*θ*) describes the relation between resistance and reactance:

(3) $$\theta =t\,a\,n^{-1}\,\frac{Z_{i}}{Z_{r}}$$

Finally, the impedance modulus or amplitude is given by:

(4) $$\left|Z\,\left(\omega \right)\right|=\sqrt{Z_{r}^{2}+Z_{i}^{2}}$$

Impedance signals from individual cells are usually analyzed in amplitude vs. phase scatterplots (Figure 1C). At low frequencies, the impedance amplitude is reflecting the particle volume and the position of the particle when moving through the electric field applied in the microchannel. At higher frequencies, the dielectric properties of the cell membrane can be interrogated. Therefore, cell sizing and counting applications are usually done at a low measurement frequency, while viability tests are typically performed at higher frequencies.

After initial testing at various frequencies, analyses were performed at 2 MHz and 8 MHz. A chip with a 120 μm × 120 μm sensing channel was used with electronic parameters adjusted as follows: the modulation gain was 0.7 V per frequency and the *trans-*impedance amplification was 8–16-fold, while the triggering level (i.e., the threshold separating signal from noise) was set to 0.05 V. A second chip with an 80 μm × 80 μm microchannel was used with a modulation gain of 0.35 V, 8–16-fold *trans-*impedance amplification and 0.07 V triggering level.

### Plant Material

In the first place, the ability of IFC to identify different levels of hazelnut pollen viability was tested using samples from the cultivar “Camponica” (from here onward: “Camponica UNITO”) collected at the experimental station of the University of Turin (Chieri, Piedmont – Italy. Latitude 45°02′27.25″ N; longitude 7°50′00.85″ E; altitude 340 m a.s.l.).

Secondly, to study hydration dynamics over time, hazelnut pollen was collected from wild and cultivated genotypes growing in orchards of the Piedmont region (Italy).

Finally, pollen viability was evaluated during blooming on 33 hazelnut cultivars situated in the experimental field “Le Cese” (Caprarola, Latium – Italy. Latitude 42°20′00″ N; longitude 12°11′00.0″ E; altitude 570 m) and on two wild hazelnut accessions growing in the surrounding vegetation (see Table 1 for a comprehensive list). Two additional pollen samples of “Nocchione” cultivar (Stelliferi A6 and F6) were collected from a nearby commercial hazelnut orchard (Nepi, Latium – Italy. Latitude 42°16′00.0″ N; longitude 12°17′00.0″; altitude 275 m). Samples were collected in January 2019 from fully elongated catkins ready to shedding pollen, either directly on plants or after overnight drying of flowers at room temperature. Pollen samples were partially dehydrated in sealed boxes with silica gel before storage at −20°C. The provenance of all the analyzed samples and the amount and distribution of catkins for each analyzed genotype are supported in Table 1.

**TABLE 1:** Amount (high; medium; low) and distribution (apical; uniform) of catkins on plants, average pollen viability and sterility (%) and relative standard deviation (sd) for 33 cultivated and two wild hazelnuts.

Cultivar	Catkins		Average sterility	Average viability	Kmc	Hc
	Amount	Distribution	(%)	(%)		
*Apolda*	Very high	Uniform	7.79 ± 0.62	89.05 ± 0.73	1	1
*Avellana Speciale*	Medium	Apical	39.89 ± 2.34	59.31 ± 2.35	3	2
*Barcelona*	Low	Apical-uniform	46.07 ± 1.57	53.35 ± 1.63	3	2
*Barrettona*	Very high	Uniform	41.46 ± 4.37	57.28 ± 4.36	3	2
*Camponica*	Medium	Apical	46.49 ± 4.16	53.01 ± 4.18	3	2
*Carrello*	Medium	Uniform	42.2 ± 9.9	56.59 ± 10.34	3	2
*Closca Molla*	Medium	Apical	55.72 ± 1.16	41.32 ± 1.09	4	2
Comen	Medium	Apical	44.18 ± 2.77	54.72 ± 2.81	3	2
*Comune di Sicilia*	High	Apical	58.81 ± 3.56	39.62 ± 3.43	4	2
*Cosford*	Very high	Uniform	13.93 ± 1.43	83.63 ± 1.37	2	1
*Daviana*	Medium-high	Apical	13.56 ± 0.67	84.19 ± 0.6	2	1
*Ennis*	High	Apical	3.76 ± 0.43	94.41 ± 0.56	1	1
*Fructo Rubro*	Medium	Apical	53.31 ± 2.17	42.32 ± 2.16	4	2
*Gironell*	Medium	Apical	3.37 ± 0.17	94.43 ± 0.21	1	1
*Grifoll*	Low	Apical	15.05 ± 1.5	82.14 ± 1.53	2	1
*Grossal*	Medium	Apical	3.52 ± 0.18	94.86 ± 0.31	1	1
*Gunslebert*	High	Apical	4.35 ± 0.11	94.56 ± 0.13	1	1
*Karidaty*	Medium	Apical	12.06 ± 0.27	86.10 ± 0.32	2	1
*Minnolara*	Medium	Uniform	42.11 ± 1.59	57.15 ± 1.56	3	2
*Negret*	High	Uniform	51.87 ± 2.27	45.2 ± 1.99	4	2
*Nocchione*	High	Uniform	51.89 ± 4.35	47.04 ± 4.44	4	2
*Nocchione “Stelliferi A6”*	High	Apical-uniform	42.48 ± 0.9	55.52 ± 0.83	3	2
*Nocchione “Stelliferi F6”*	High	Apical-uniform	44.11 ± 1.98	54.21 ± 2.08	3	2
*Nociara*	High	Apical	49.99 ± 4.31	49.09 ± 4.4	4	2
*Nostrale*	High	Uniform	42.3 ± 1.18	56.65 ± 1.24	3	2
*Pallagrossa*	High	Uniform	6.46 ± 2.45	91.22 ± 2.43	1	1
*Piazza Armerina*	High	Uniform	57.13 ± 4.46	41.96 ± 4.41	4	2
*Racinante*	High	Apical	37.08 ± 1.82	62.1 ± 1.89	3	2
*Riccia di Talanico*	Medium	Apical	48.27 ± 4.22	50.32 ± 4.21	3	2
*Santa Maria del Gesù*	Medium	Uniform	45.52 ± 1.41	53.96 ± 1.43	3	2
*Tombul*	Low-medium	Apical	3.88 ± 0.27	94.72 ± 0.34	1	1
*Tonda Bianca*	Medium	Apical	0.96 ± 0.08	98.13 ± 0.08	1	1
*Tonda di Giffoni*	High	Uniform	73.58 ± 3	23.96 ± 2.8	5	2
*Tonda Romana*	High	Uniform	45.51 ± 9.3	51.17 ± 9.31	3	2
*Vermellet*	Medium	Uniform	46.11 ± 5.8	53.27 ± 5.76	3	2
*Wild Type 1*	Medium-high	Apical	2.64 ± 0.12	97.02 ± 0.16	1	1
*Wild Type 2*	Medium-high	Apical	1.85 ± 0.3	97.81 ± 0.33	1	1

*Kmc, cluster membership following k-means clustering; Hc, cluster membership based on hierarchical clustering.*

### Preparation of Pollen Samples and Impedance Analysis

To validate the experimental setup, a small amount of pollen from “Camponica UNITO” cultivar was dispersed in the analysis buffer AF6 buffer (Amphasys, Switzerland) and half of the sample was left inactivating by heat in boiling water for 30 min. Untreated and heat-inactivated pollen were mixed in 1:0, 3:1, 1:1, 1:3, and 0:1 ratios before the analysis using the 80 μm × 80 μm channel chip. Two samples, three replicas and 20′000 pollen grains per replica were analyzed at every mixing ratio.

Pollen rehydration dynamics were monitored by placing a small amount of pollen for each hazelnut genotype (Table 1) at the bottom of sealed Eppendorf tubes with imbibed hydrophilic cotton inside, before suspending pollen with measurement buffer AF6 (Amphasys, Switzerland). Impedance was monitored using the 120 μm × 120 μm microchannel chip every 30 min from time 0 up to 7 h. The stability in the buffer of rehydrated and non-rehydrated pollen from the same hazelnut genotype was also monitored for 1.5 h every 15 min. A total of nine samples of 10′000 pollen grains each were measured per sample.

Finally, pollen viability was assessed on the 33 cultivated and two wild hazelnut genotypes (Table 1). For this latter experiment, one sample comprising three replicas of 20′000 pollen grains per replica was analyzed for each hazelnut genotype using the chip with a 120 μm × 120 μm microchannel. Apart from the samples used for the rehydration monitoring, all pollen was left pre-hydrating for 6 h in improvised humid chambers as described above. Before each analysis, some pollen was dispersed in the analysis buffer and left to stabilize for 10 min. All impedance measurements were carried out in the AF6 buffer (Amphasys, Switzerland), a proprietary buffer containing mineral salts and sugars with conductivity and osmolarity suitable for hazelnut pollen grains. Samples were always filtered with 50 μm strains (Sysmex, Italy) before the analysis.

### IFC Data Analysis

Pollen populations were identified on scatterplots using fixed polygon gates for each hazelnut cultivar and genotype. In the experiment of pollen hydration monitoring over time, gates were placed when populations were best separated from one another and then applied to all the other time steps. The gating procedure was performed using AmphaSoft 2.1.6 (Amphasys, Switzerland). For further investigation, measurement data were exported in CSV databases and analyzed in the R environment (R Core Team, 2019). The experimental setup was validated with linear regression models and adjusted coefficient of determination ($R_{\text{adj}}^{2}$). Pollen viability of the 33 cultivated and two wild hazelnut genotypes was analyzed with boxplots. Pollen sterility was grouped using either package “Ckmeans.1d.dp” (Wang and Song, 2011) by fast univariate k-means clustering and the number of clusters estimated by the normalized Bayesian Information Criterion (n/BIC), or package “fastcluster” for fast hierarchical clustering and the number of clusters estimated by Silhouette (Rousseeuw, 1987) and Gap statistic (Tibshirani et al., 2000). Finally, the relationship between average pollen sterility and average amplitude values of the analyzed hazelnut genotypes was inspected through linear regression models and Pearson correlations (*R*).

### Pollen Staining and Image Analysis for IFC Validation

The viability of “Camponica UNITO” pollen was assessed employing the dye exclusion principle. Rehydrated pollen (Amphasys, Switzerland) was heat-treated and mixed with viable pollen in buffer AF6 in 1:0, 3:1, 1:1, 1:3, and 0:1 ratios. Pollen was centrifuged at 5000 rpm for 5 min and the supernatant was discarded. Staining was carried out in Brewbaker and Kwack (BK) medium (Brewbaker and Kwack, 1963) with the addition of 0.5% acid fuchsine. Dead pollen is unable to exclude the stain due to a damaged outer membrane and is colored dark pink, while viable pollen remains unstained. Imaging was performed in bright-field microscopy at 4 × employing a Leitz Diaplan microscope connected to a Leica DFC420 camera with a 5 MP Sony ICX452 CCD sensor, on two samples and three replicas each. Image segmentation and object detection were performed using CellProfiler software (McQuin et al., 2018), coupled with CellProfiler Analyst (Dao et al., 2016) for automated classification of dead, viable and sterile pollen grains using a supervised random forest algorithm. 4241 pollen grains per replica were counted on average.

## Results

### Evaluation of Pollen Viability and Sterility With Impedance Flow Cytometry

**FIGURE 2:**
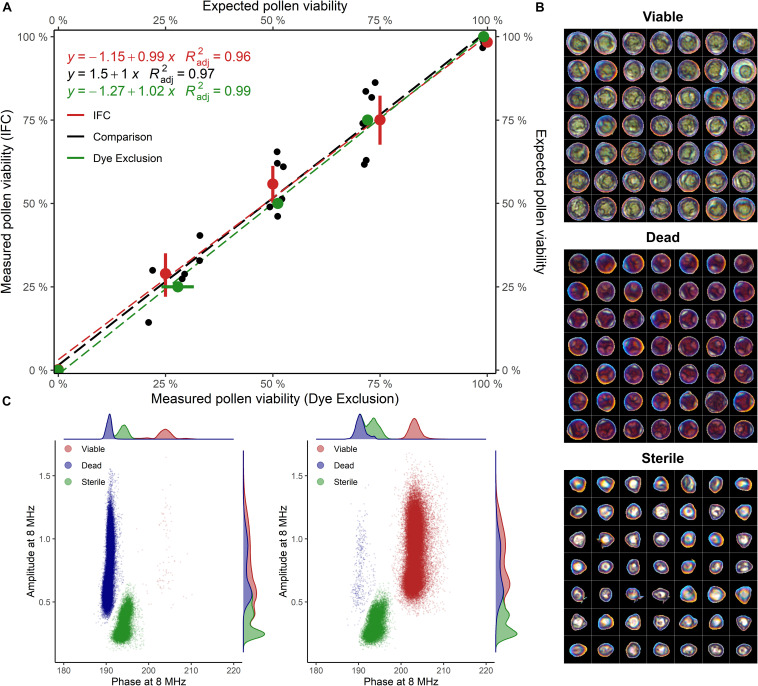
**(A)** Viability of untreated and heat-inactivated pollen grains mixed in different proportions (1:0, 3:1, 1:1, 1:3, and 0:1), evaluated with IFC and image analysis. Samples of cv “Camponica UNITO” were used in this experiment. For percentage computation, sterile pollen was excluded. $R_{\text{adj}}^{2}$ = adjusted coefficient of determination. **(B)** Viable, dead, and sterile pollen grains stained with acid fuchsine and segmented from images **(C)** Phase-amplitude scatterplots with per-axis kernel densities of heat-inactivated (100% dead) and untreated (100% viable) “Camponica UNITO” pollen. Data from one sample (60′000 grains) was used for scatterplot generation.

Staining of pollen from cv. “Camponica UNITO” paired with image analysis discriminated between three pollen types showing different morphologies and dimensions. Pollen grains with a regular morphology were classified either as viable (unstained), with an average maximum Feret diameter of 38 ± 4 μm, or dead (dark-pink), with an average maximum Feret diameter of 34 ± 3 μm. An additional class of smaller (27 ± 3 μm), anomalous pollen grains, characterized by no staining and the absence of the typical internal structures of hazelnut pollen (Figure 2B), was considered sterile.

IFC of “Camponica UNITO” at 8 MHz using the 80 μm × 80 μm microchannel chip showed that two populations of hazelnut pollen changed their size according to the proportion between treated and untreated samples (Figure 2C). The first population was considered coming from viable pollen as it was characterized by higher average phase and amplitude values (200° ± 1.35° and 0.9 ± 0.23), while the second population was considered coming from dead pollen as it was differentiated by an average lower phase (188° ± 0.72°) and similar amplitude (0.9 ± 0.25). A third population having a lower average amplitude (0.364 ± 0.11) and an intermediate average phase (190° ± 1.1°) was detected invariably throughout the experiment (Figures 2B,C). This last group was considered sterile pollen, as smaller amplitude is indicative of smaller pollen size, whereas the constant proportion throughout the experiment reflects the absence of cellular compartments that could be damaged by the heat treatment.

In the comparison between the two methods, both image analysis and IFC were able to identify different pollen viability levels in cv. “Camponica UNITO” with good accuracy ($R_{\text{adj}}^{2}$ = 0.99 and $R_{\text{adj}}^{2}$ = 0.96). Moreover, the two methods strongly agreed ($R_{\text{adj}}^{2}$ = 0.97) as shown in Figure 2A.

### Hazelnut Pollen Hydration Dynamics and Best Rehydration Time Window

**FIGURE 3:**
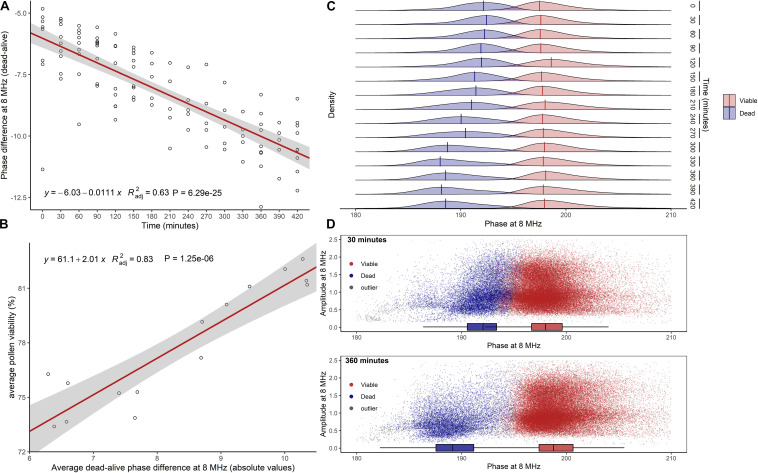
Effect of pollen hydration over time (minutes) explored by IFC at 8 MHz. **(A)** Difference between average phase values of dead and viable pollen populations. In red, linear regression fitted to data. **(B)** Linear relationship between average pollen viability at each hydration step and average absolute difference between viable and dead phase values. **(C)** Kernel densities and global modes estimated for dead and viable phases at each time step. **(D)** Scatterplots of viable and dead pollen (sterile pollen was not considered) at 30 min (top) and 360 min (bottom) of hydration. $R_{\text{adj}}^{2}$ = adjusted coefficient of determination.

Impedance measurements of hazelnut pollen at 8 MHz and with the 120 μm × 120 μm channel chip in saturated air at room temperature showed that the separation between viable and dead phase values increased with increasing hydration time (Figure 3A). A significant positive relationship ($R_{\text{adj}}^{2}$ = 0.83, *P* ≤ 0.0001) was found between changes in average pollen viability (Figure 3A) and changes of absolute average difference among viable and dead phase values (Figure 3B). Viable pollen stabilized at mode phase values of 198°, while dead pollen moved from mode values of 192° at 0′ to 188° after 5.5 h (Figure 3C). This phenomenon resulted in a better separation of viable and dead pollen populations after 6 h of hydration treatment (Figure 3D). Following the progressive decrease in dead pollen volume, also the amplitude of this population slightly but significantly decreased throughout hydration (Supplementary Figure 1A). Phase and amplitude values of sterile pollen remained constant over the hydration experiment (Supplementary Figures 1B,C). Finally, the viability of pollen from the same hazelnut genotype remained stable in buffer AF6 if rehydrated for 6 h, but quickly dropped if not rehydrated (Supplementary Figure 1D).

### Pollen Quality of Selected Hazelnut Genotypes

**FIGURE 4:**
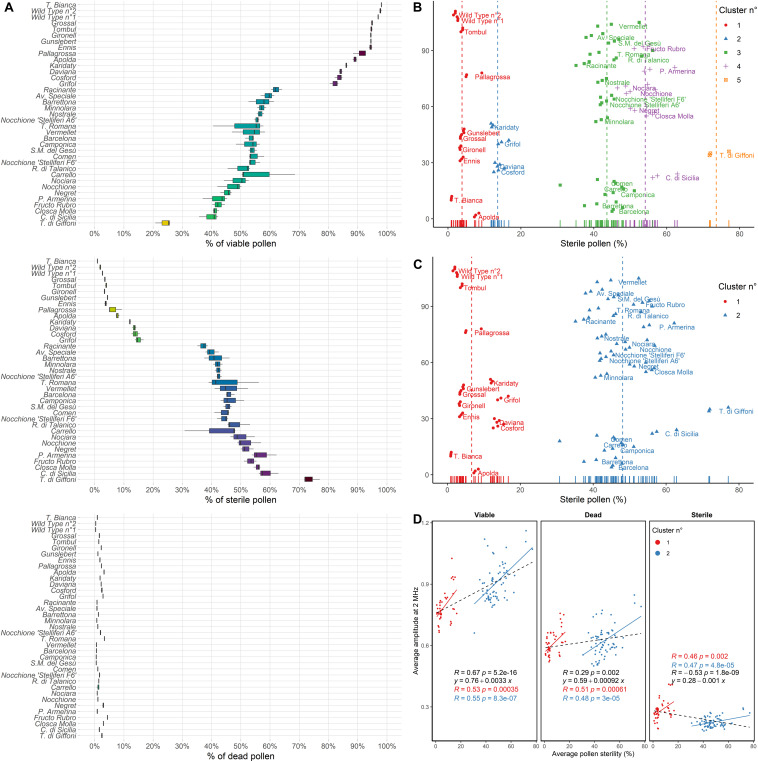
**(A)** Percent (%) of viable, sterile and dead pollen of 33 cultivated and two wild hazelnuts sampled in the Latium region (Italy) ordered by the percent of viable pollen. **(B)** Average pollen sterility clustered by k-means clustering following the number of clusters defined by the normalized Bayesian Information Criterion (BIC/n) and **(C)** Gap statistic or Silhouette methods. Dot colors and shapes change following cluster membership. Each dot corresponds to one sample replica. Labels are placed beside per-cultivar average values. **(D)** Pearson correlations (R) and linear regressions of average pollen sterility (%) and average amplitude at 2 MHz of the analyzed hazelnut genotypes for viable, dead and sterile pollen. Colors correspond to cluster membership as assessed by gap statistic or Silhouette and hierarchical clustering. In black general linear regressions and Pearson correlations.

Pollen viability of various hazelnut genotypes measured at 8 MHz using a 120 μm × 120 μm channel microchip after 6 h of pre-hydration treatment is shown in Figure 4A. Many of the most popular hazelnut cultivars, such as “Tonda di Giffoni,” “Comune di Sicilia,” and “Negret” produced a copious amount of sterile pollen mainly responsible for lower viability. On the contrary, “Tonda Bianca,” “Grossal,” “Tombul,” “Gironell,” “Gunslebert,” “Ennis,” “Pallagrossa,” “Apolda” cultivars and the two wild hazelnut plants, all produced low quantities of sterile pollen (Figure 4A).

Furthermore, the cultivars tested showed different amounts and distribution in the crown of male inflorescences (catkins), as reported in Table 1. One group of accessions, namely “Apolda,” “Barrettona,” “Carrello,” “Cosford,” “Minnolara,” “Negret,” “Nocchione,” “Nostrale,” “Pallagrossa,” “Piazza Armerina,” “Santa Maria del Gesù,” “Tonda di Giffoni,” “Tonda Romana,” and “Vermellet,” were distinguished for their homogeneous distribution of catkins throughout the tree canopy, while the other accessions had a high apical incidence of catkins. Referring to catkin amounts on the plant, the cultivars “Apolda,” “Barrettona,” and “Cosford” had the highest quantity of elongated catkins during blooming, contrary to “Barcelona,” “Grifoll,” and “Tombul” that showed the lowest amount.

Hierarchical clustering based on silhouette and gap statistic methods identified two main groups of hazelnut genotypes, with average pollen sterility of 6.7% and 48.1%, respectively (Figure 4C). However, five groups were found using n/BIC and k-means clustering, characterized by 3.9%, 13.6%, 43.6%, 54.1%, and 73.6% pollen sterility (Figure 4B). The analysis of impedance at 2 MHz showed a cluster-dependent relationship between average pollen sterility and average amplitude (i.e., average size) in viable, dead and sterile pollen of the analyzed hazelnut genotypes. Hazelnut genotypes that displayed higher pollen sterility also tended to produce viable pollen with higher average size and sterile pollen with lower average size (Figure 4D). Gated scatterplots for each hazelnut genotype are shown in Supplementary Figure 2.

## Discussion

The analysis of single cells in flow cytometry has enabled a wide range of research opportunities in plant biology (Doležel et al., 2007). Despite this long tradition, the application of flow cytometry that relies on fluorescent labeling to the analysis of intact pollen has been hampered by some intrinsic characteristics of the pollen grain, such as the highly variable size and wall thickness and composition (Suda et al., 2007). As a consequence, flow cytometry was mainly relegated to the study of pollen ploidy, using pollen with the outer wall removed (Kron and Husband, 2012, 2015; Kron et al., 2014). Only very recently, flow cytometry using probes for reactive oxygen species was showcased for the evaluation of pollen viability (Luria et al., 2019). This left space for the development of alternative techniques to study pollen performance traits using either image analysis (Mudd and Arathi, 2012; Tello et al., 2018; Ascari et al., 2020) or impedance flow cytometry (Heidmann et al., 2016; Heidmann and Di Berardino, 2017). IFC in particular provided the opportunity of measuring a high number of pollen grains and distinguished well between viable and dead pollen in high-throughput without requiring long sample preparation and staining procedures (Heidmann et al., 2016).

In this study, different populations of viable, heat-inactivated (i.e., dead) and sterile pollen grains were identified in two cultivated hazelnuts (Figure 2). Dead and viable pollen were characterized by lower and higher average phase angles, respectively, reflecting a different membrane capacitance. The loss of membrane integrity, as evidenced by lower membrane capacitance, is one of the footprints of pollen death (Shivanna and Heslop-Harrison, 1981).

Being partially hydrated at dispersal, hazelnut pollen should be particularly sensitive to water loss and, conversely, could benefit from elevated ambient relative humidity (Nepi et al., 2001; Firon et al., 2012). Hazelnut dead pollen dimensions decreased constantly during hydration (Supplementary Figure 1A) likely following a progressive decline in membrane integrity. It was also shown that immediately after the storage period, part of the hazelnut pollen grains presented phase angles that classified them neither as viable nor dead. Instead, the signal was similar to that of tomato pollen partially deactivated by temperature treatments above 37°C (Heidmann et al., 2016). After around 6 h of pre-hydration, the same population reached phase values of heat-inactivated pollen, while overall pollen viability slightly increased at the same time (Figure 3). A possible interpretation is that a fraction of the partially inactivated pollen was able to rehydrate well and appeared viable after 6 h. The remaining part was likely too damaged and not able to recover an intact membrane. The presence of this population could be explained by an inappropriate dehydration treatment before storage, but also by natural factors, like developmental asynchrony and temperature (Harsant et al., 2013; Carrizo García et al., 2017). Interestingly, measured pollen viability depended on pollen hydration ($R_{\text{adj}}^{2}$ = 0.83, *P* ≤ 0.0001). Rehydration also ensured the complete stability of pollen viability in the buffer over time (Supplementary Figure 1D). Heslop-Harrison et al. (1986) found that hazelnut pollen germination was able to benefit from long pre-hydration treatments. Controlled re-hydration not only ensures the recovery of the outer membrane (Shivanna and Heslop-Harrison, 1981) but is also a key factor for pollen metabolic reactivation and pollen tube emergence on the stigma (Edlund et al., 2004). A mechanosensitive ion channel (MSL8) of the plasma membrane in the partially dehydrated *Arabidopsis* pollen was recently characterized (Hamilton et al., 2015). Interestingly, plants with MSL8 were able to survive osmotic stress in distilled water, while those lacking the channel soon died after the treatment. This observation implies that partially dehydrated pollen could have developed an active mechanism to endure hypo-osmotic shocks even without earlier pre-hydration. IFC could be a valuable technique for investigating the behavior of pollen in different osmotic environments further.

Impedance flow cytometry was also employed to detect different degrees of sterility in hazelnut plants. Populations of sterile pollen were characterized by lower average amplitudes indicative of lower average dimensions (Figure 4D). As shown by the fuchsine staining, sterile pollen grains have a subtriangular shape, lower dimensions and appear emptied of the cytoplasm content (Figure 2). Most of the analyzed hazelnut cultivars produced copious sterile pollen (Figure 4D). Other authors also reported the presence of anomalous, sterile pollen in hazelnut (Mckay, 1965; Potenza et al., 1994; Frenguelli et al., 1997). Plant male sterility can result from gametophytic mutations (Sari-Gorla et al., 1996; Procissi et al., 2001), chromosome rearrangements (Nonomura et al., 2004; Stathos and Fishman, 2014), extreme temperatures or drought regimes (Sheoran and Saini, 1996; Rieu et al., 2017), irradiations (Vazquez and Sanche-Monge, 1987), and hybridization in cultivated plants (Long et al., 2008). Different authors also reported a higher incidence of deformed or anomalous pollen in natural populations of *Quercus*, *Betula*, and *Crataegus* interspecific hybrids (Karlsdóttir et al., 2008; Wrońska-Pilarek et al., 2013, 2016). Moreover, it was demonstrated that even plant age can influence the production of anomalous pollen (Aizen and Rovere, 1995). Therefore, it would be deceptive to infer the cause of hazelnut pollen sterility solely based on the collected data. Nevertheless, the neat separation between at least one group of highly sterile cultivars and a second group with lower sterility values (Figure 4C) supports the findings of heterozygous translocations in various hazelnut cultivars (Salesses, 1973; Salesses and Bonnet, 1988; Marinoni et al., 2018). Some of the cultivars affected by pollen sterility are also of strong economic importance for their good yield and fruit organoleptic properties, nut traits and kernel (Cristofori et al., 2008; Solar and Stampar, 2011). Therefore, other factors such as plant vigor and female flower densities could have somewhat compensated for the lack of fertile gametes. A reciprocal translocation without an apparent effect on plant fitness and yield is also present in the barley cultivar Albacete (Farré et al., 2012).

Finally, the relationship between pollen sterility and pollen dimensions was investigated (Figure 4D). Strong relationships between pollen size and DNA content were found in different plant species (Lavia et al., 2011; Pécrix et al., 2011; De Storme et al., 2013; Srisuwan et al., 2019). Extrapolating data from De Storme et al. (2013) we can compute an average increase of 12 ± 3.5% in pollen volume at each ploidy level in *Arabidopsis*. For hazelnut, viable pollen is 15% bigger in highly sterile cultivars than in cultivars with low sterility (Figure 4D). Therefore, if ploidy scales equally with volume and amplitude, we would have detected reduced haploid (smaller) and unreduced diploid (bigger) gametes. Non-reductional meiosis is the most frequent pathway to unreduced gamete formation, though not all the produced gametes are equally affected, with both environment and genetic mutations influencing the frequency of unreduced pollen (Pécrix et al., 2011; De Storme and Geelen, 2013). Alternatively, unreduced pollen can be found in polyploid plants, a circumstance that should be excluded for hazelnut as different genotypes have been showing a diploid genome (Guo et al., 2009; Falistocco and Marconi, 2013). The reciprocal translocations purportedly causing pollen sterility in hazelnut are more consistent with the formation of aneuploid (i.e., with supernumerary chromosomes) gametes following secondary patterns of chromosome segregation which, however, are considered to be rarely compatible with viability (Scriven et al., 1998; Gardner and Sutherland, 2012).

Studying microsporogenesis in different Corylus species, Woodworth (1929) reported the presence of cytomixis, an exchange of bivalents or even entire nuclei between pollen mother cells during microsporogenesis (Mursalimov and Deineko, 2018). This phenomenon has been observed in combination with polyploidy and pollen sterility (Negrón-Ortiz, 2007; Singhal and Kumar, 2008; Pécrix et al., 2011; Kumar et al., 2013; Reis et al., 2016) and is thought to be one of the cytological processes occurring during unreduced pollen formation (Falistocco et al., 1995; Mursalimov and Deineko, 2015), even if there is not enough evidence to support its role as a supplementary mechanism for genetic recombination (Mursalimov and Deineko, 2018). Taken as a whole, the lack of additional data on hazelnut microsporogenesis hinders drawing a definite picture around the rationale behind the observed increase in pollen size and point to the need for a deeper study of meiosis during hazelnut pollen development in low and high sterile cultivars.

As a closing remark, IFC turned out to be strikingly useful for the label-free characterization of hazelnut pollen in a high throughput, allowing the fast identification of hazelnut cultivars with high male sterility. Moreover, its versatility holds great promise for numerous research applications in hazelnut pollen biology and pollination ecology and has the potential to simplify haploid plant production for hazelnut breeding purposes (Gniech Karasawa et al., 2016), as demonstrated by recent studies on wheat (Canonge et al., 2020).

## Conclusion

Our results demonstrate that IFC technology is well suited for the extensive characterization of hazelnut pollen traits. We showed how hazelnut pollen responds to prolonged hydration times through changes in the impedance signal. We also detected high pollen sterility in many cultivated hazelnut genotypes possibly linked to chromosome translocations. Finally, we observed an increase in pollen dimensions in highly sterile cultivars. Additional inspection of pollen development, especially in the meiotic phase, is required to explain the increase in pollen volume associated with pollen sterility. Moreover, further investigation is needed to confirm the occurrence of chromosome translocations and identify the involved chromosomes. This is especially important to enhance the choice of suitable pollinators for the most popular hazelnut cultivars, particularly when this cultivated species is introduced in new growing areas.

## Data Availability Statement

The original contributions presented in the study are included in the article/Supplementary Material, further inquiries can be directed to the corresponding author.

## Author Contributions

LA, TD, EH, and VC conceived and designed the experiments. LA, CS, EH, MD, FM, and SK performed the experiments and acquired and analyzed the data. LA performed statistical analyses and drafted the manuscript. VC, RB, and CS critically revised the manuscript. All authors contributed to the article and approved the submitted version.

## Conflict of Interest

TD and EH were employed by the Ferrero Trading Lux (Luxembourg), MD and SK were employed by the Amphasys AG (Switzerland). The Hazelnut Company division of Ferrero Trading Lux (Luxembourg) financed the first author’s Ph.D. grant and coordinated the research. The funder was not involved in data analysis and interpretation, nor in the decision to submit this article for publication. Amphasys AG supplied the cytometer used in the study and provided expert assistance in data interpretation. The remaining authors declare that the research was conducted in the absence of any commercial or financial relationships that could be construed as a potential conflict of interest.
