# Supplementary material for: Hazelnut Pollen Phenotyping Using Label-Free Impedance Flow Cytometry
Source: Front Plant Sci. 2020 Dec 8;11:615922. doi: 10.3389/fpls.2020.615922 (PMC7753158; doi:10.3389/fpls.2020.615922)
Supplement: Supplementary file 1 [file Data_Sheet_1.docx]

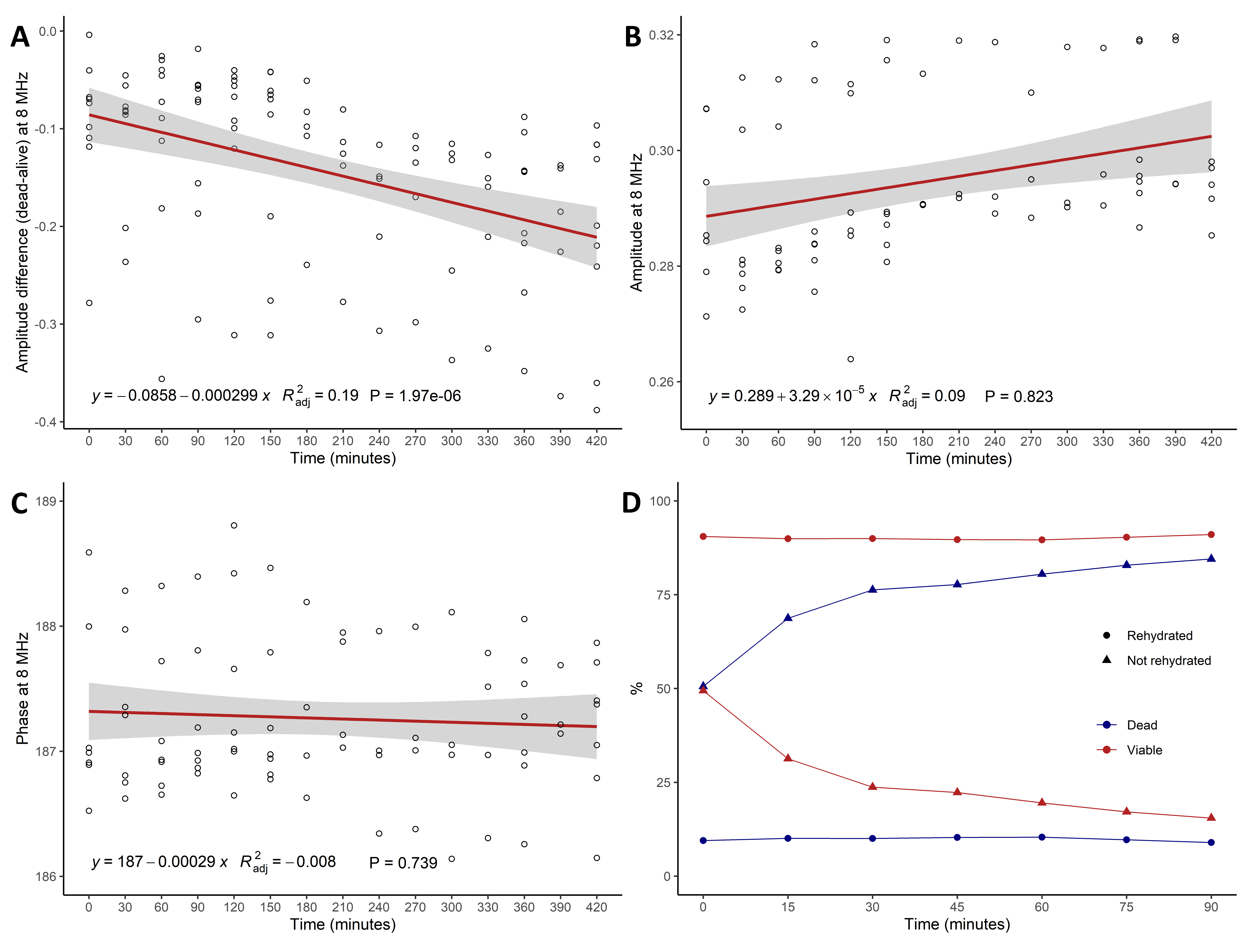


Fig. S1. Effect of hydration treatment on (**A**) difference between the amplitude of viable and dead pollen at 8 MHz. Effect of hydration treatment on (**B**) amplitude and (**C**) phase values of sterile pollen at 8 MHz. Fitted linear regression in red. (**D**) Stability of pre-hydrated and non-pre-hydrated pollen from the same hazelnut genotype in buffer AF6. Point shapes and colors refer to hydration treatments and pollen states respectively. $R_{adj}^{2}$ = adjusted coefficient of determination.
