# Supplementary material for: Hazelnut Pollen Phenotyping Using Label-Free Impedance Flow Cytometry
Source: Front Plant Sci. 2020 Dec 8;11:615922. doi: 10.3389/fpls.2020.615922 (PMC7753158; doi:10.3389/fpls.2020.615922)
Supplement: Supplementary file 2 [file Data_Sheet_2.docx]

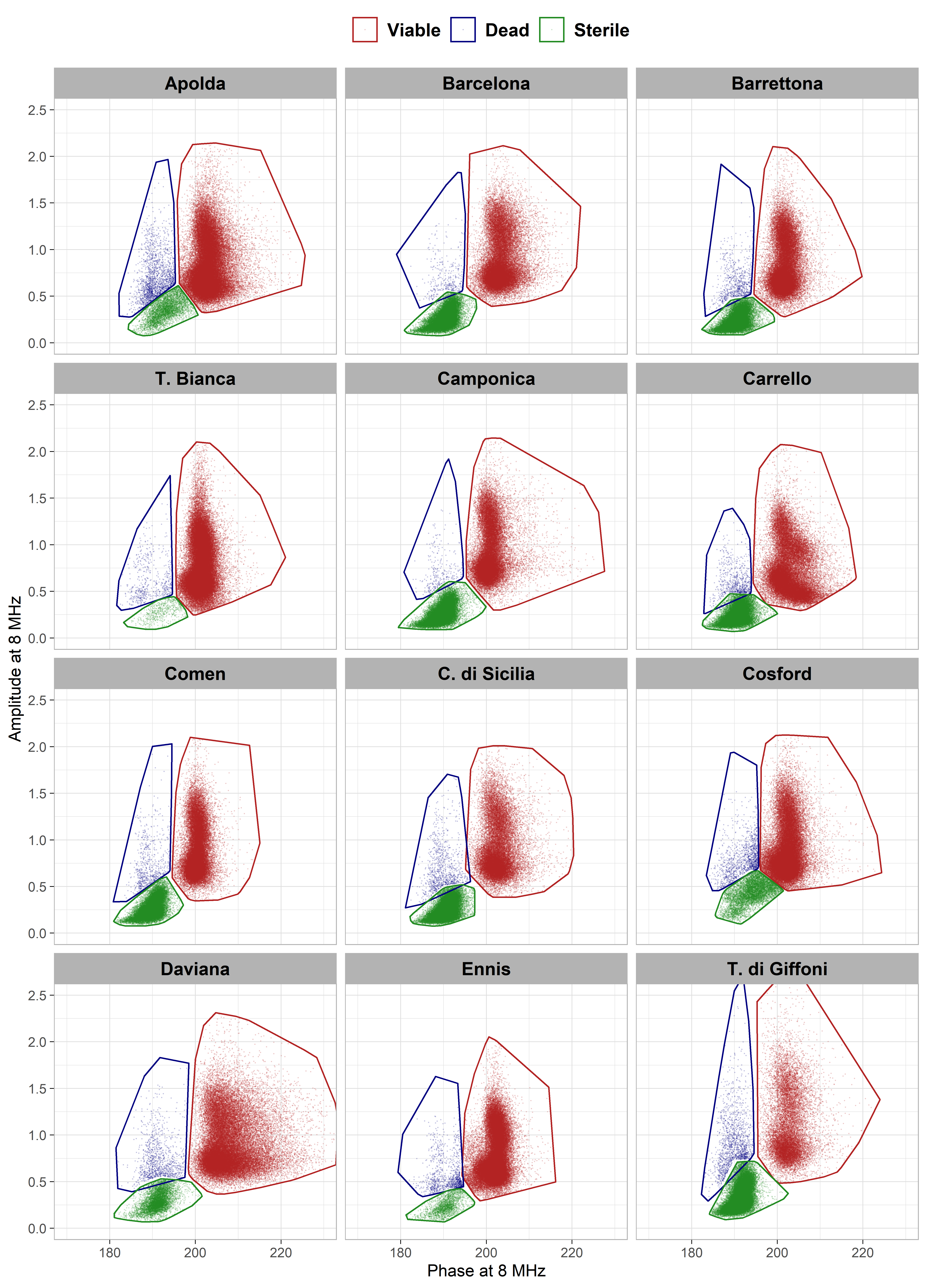


Fig. S2. Gated phase vs amplitude scatterplots for 33 cultivated and two wild hazelnuts analyzed at 8 MHz on a chip provided with a 120 µm × 120 µm microchannel.


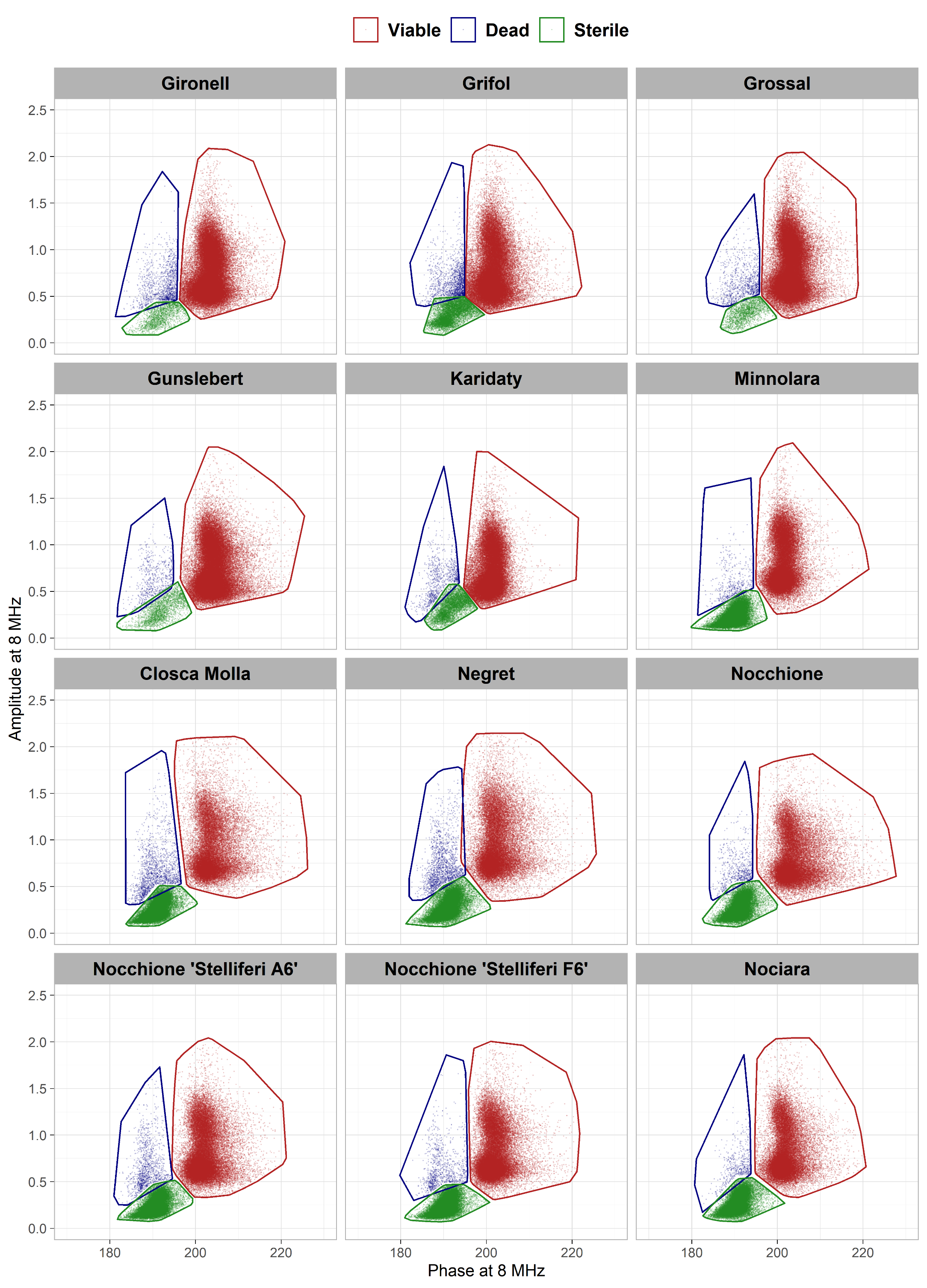


Fig. S2 (continue). Gated phase vs amplitude scatterplots for 33 cultivated and two wild hazelnuts analyzed at 8 MHz on a chip provided with a 120 µm × 120 µm microchannel.


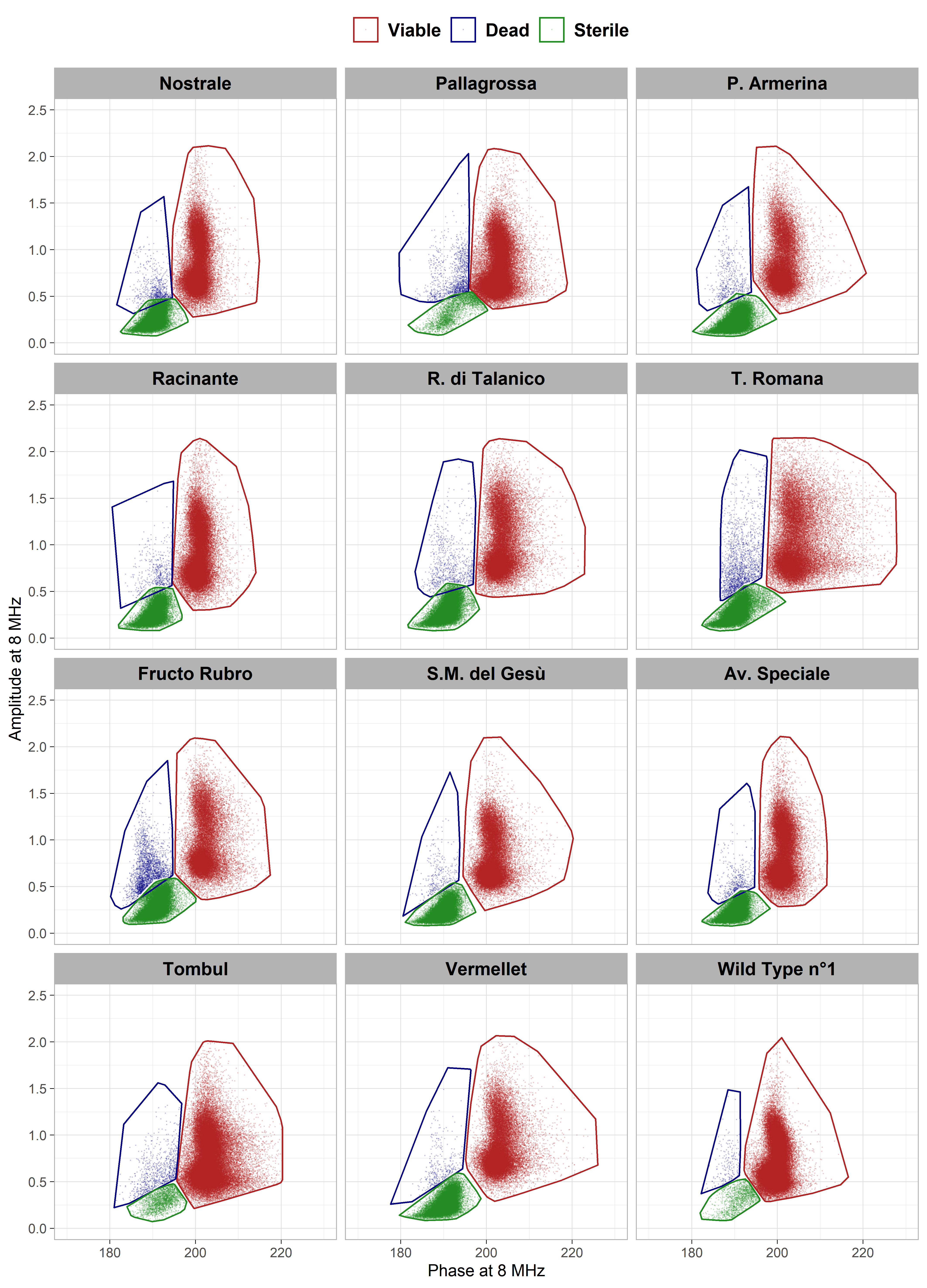


Fig. S2 (continue). Gated phase vs amplitude scatterplots for 33 cultivated and two wild hazelnuts analyzed at 8 MHz on a chip provided with a 120 µm × 120 µm microchannel.


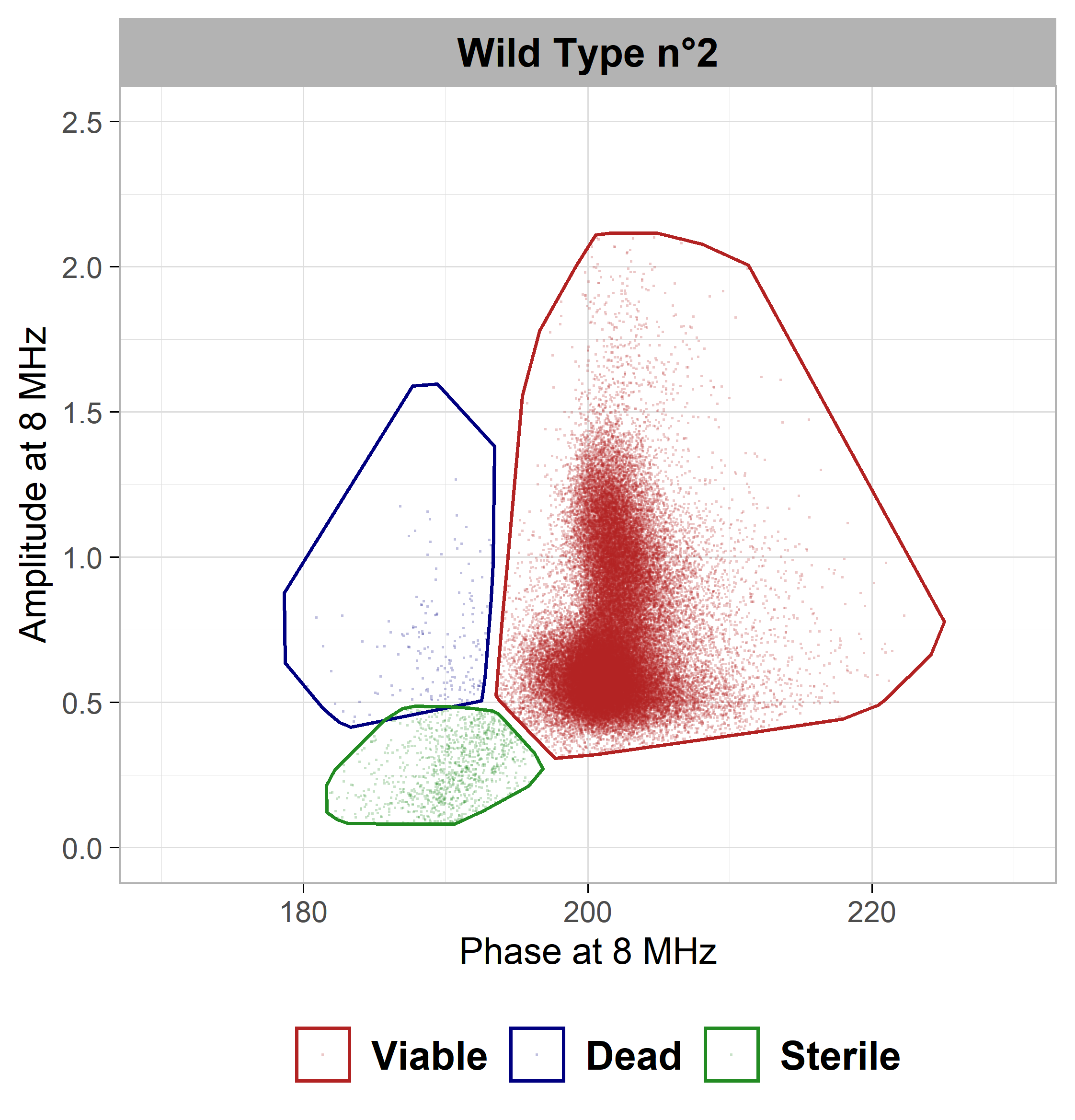


Fig. S2 (continue). Gated phase vs amplitude scatterplots for 33 cultivated and two wild hazelnuts analyzed at 8 MHz on a chip provided with a 120 µm × 120 µm microchannel.
